# Domestic

**Published:** 1842-01-29

**Authors:** 


### DOMESTIC.

*Milky Cyst in the Mamma.*—We extract from the notes of a Clinical Lecture of Professor Parker, in the theatre of the College of Physicians and Surgeons of New York, reported in the N. Y. Medical Gazette, January 19, 1842, the following remarkable case:

"This patient is about thirty, apparently in good health, has always worked hard, now has an infant nine months old, which she has nursed regularly from both breasts; the right is enlarged immensely. The patient states that the breast began to increase in size in July last, that it has gone on steadily augmenting, but without pain or febrile symptoms. The tumour is not now painful, but causes considerable inconvenience by its size. The nipple is natural, so is the skin; the blood-vessels of the breast are somewhat enlarged. On looking at this case our first idea would be, that we had hypertrophy of the breast, or else a tumour situated behind the gland pushing it forward; but on examining carefully we find distinct fluctuation; the swelling is therefore not solid but fluid. What then is the probable nature of that fluid? We have no reason to think that it is pus; having no evidence that the inflammation essential to the formation of pus has at any time existed. The tumour may be from hydatids, or from a collection of milk distending an obstructed lactiferous tube, though its very large size renders the latter supposition improbable.

As the case is obscure, I shall first perform an explorative operation, and push in a sharp pointed bistoury. '(This was done, and milk escaped. The true character of the tumour being now evident, a trochar was pushed in and milk gushed forth in a free stream; it was collected in basins; it was perfectly sweet and apparently quite unchanged; being measured after lecture, was found to be three full quarts. This milk, as wewere afterwards informed, being allowed to stand 24 hours, gave a good quantity of cream.)' The nature of this case is now evident enough; it is a very remarkable one; I have never seen a tumour of this character approach the size of the one we have here; they are generally not larger than an orange. Every now and then lacteal fistulæ result from the opening of these sacs; in that case milk will flow from the artificial opening every time the child nurses, and the only remedy is to wean him; the breast then ceasing to secrete milk, the opening will heal. I shall direct this woman to call on us next Monday, that we may see the progress of this exceedingly curious case.

*Monday, Jan.* 10.

The patient with lacteal tumour again appeared in the theatre. The opening made by the trochar had healed by the first intention, and the milk had again accumulated in the breast. The organ was pendulous and somewhat pediculated; its circumference was 22 inches; fluctuation, more distinct at some points than at others, was evident all over the tumour. The trochar was again plunged in, and about two quarts of pure sweet milk drawn off. The patient was advised to wean her child."

*Compound fracture of the arm, complicated with laceration of the brachial artery.*—In the same number of the same Journal, we find a case of this severe complication, reported by Dr. Alfred C. Post. The termination was happy under such untoward circumstances, notwithstanding the unavoidable limitation of the motions of the elbow-joint:

"W. L., aged 52, was stepping from his stoop to a pump, which stood near his house, on the 16th February, 1835, the ground being at the time covered with a very smooth coating of ice, when he slipped and fell, his right elbow coming in contact with the pavement. I was passing by when he fell, and I assisted him to rise. I perceived at once that his arm was broken, and the arterial blood was flowing from it. My first impression was that he had been wounded by a fragment of a pitcher which he had in his hand when he fell; but on exposing his arm, I found that there was a small laceration of the integuments at the seat of the fracture, which was just above the condyles of the os brachii, and that arterial blood was flowing very freely from the wound. I immediately compressed the artery against the bone, above the seat of the injury, and sent a messenger in quest of Dr. Buck, who soon came to my assistance. I then requested Dr. Buck to make pressure upon the artery, while I made a free incision in the situation of the injury, including the original wound made by the fragment of the broken bone. I had some difficulty in finding the artery, in consequence of the blood which was ejected into the cellular tissue. When the vessel was exposed I perceived an extensive laceration of its coats, and I applied two ligatures, one above and the other below the laceration. The edges of the wound were brought together in the usual manner. The wound was dressed on the fifth day after the operation; there was not much union by first intention. A few days after the operation the patient became very much prostrated, with a feeble pulse, diarrhœa and vesications on the hand and fore-arm. By the use of astringents, tonics, and stimulants, these symptoms were arrested, and the threatening gangrene of the limb was prevented. The wound healed, and the bone was consolidated in about two months from the time of the injury. In consequence of the feeble vitality of the limb, the pressure of splints and bandages could not be borne, and the limb could only be supported on a trough or hollow splint, without bringing the fragments of the bone into a proper state of apposition. A considerable amount of shortening took place, and from the proximity of the fracture of the elbow-joint, the motions of that articulation were very much restrained.

The patient is still living and in good health, nearly seven years after the injury, but he has very little motion at the elbow of the affected limb."

